# Transaction of the Minnesota State Medical Society

**Published:** 1871-09

**Authors:** 


*Transactions of the Minnesota State Medical Society.*

This pamphlet contains addresses and reports, with illustrative cases, on the various branches of medicine, and will be read with interest by the profession.

